# Qualitative Approximations to Causality: Non-Randomizable Factors in Clinical Psychology

**DOI:** 10.32872/cpe.3873

**Published:** 2021-06-18

**Authors:** Michael Höfler, Sebastian Trautmann, Philipp Kanske

**Affiliations:** 1 Clinical Psychology and Behavioural Neuroscience, Institute of Clinical Psychology and Psychotherapy, Technische Universität Dresden, Dresden, Germany; 2 Department of Psychology, Medical School, Hamburg, Germany; 3 Max Planck Institute for Human Cognitive and Brain Sciences, Leipzig, Germany; Philipps-University of Marburg, Marburg, Germany

**Keywords:** causality, causal considerations, counterfactuals, directed acyclic graphs

## Abstract

**Background:**

Causal quests in non-randomized studies are unavoidable just because research questions are beyond doubt causal (e.g., aetiology). Large progress during the last decades has enriched the methodical toolbox.

**Aims:**

Summary papers mainly focus on quantitative and highly formal methods. With examples from clinical psychology, we show how qualitative approaches can inform on the necessity and feasibility of quantitative analysis and may yet sometimes approximate causal answers.

**Results:**

Qualitative use is hidden in some quantitative methods. For instance, it may yet suffice to know the direction of bias for a tentative causal conclusion. Counterfactuals clarify what causal effects of changeable factors are, unravel what is required for a causal answer, but do not cover immutable causes like gender. Directed acyclic graphs (DAGs) address causal effects in a broader sense, may give rise to quantitative estimation or indicate that this is premature.

**Conclusion:**

No method is generally sufficient or necessary. Any causal analysis must ground on qualification and should balance the harms of a false positive and a false negative conclusion in a specific context.

## Highlights

- Causal inference outside randomized, controlled experiments and trials is rare in clinical psychology, regardless of the rich methodology that has evolved in the last decades.
- The attractiveness of these new formal tools distracts from their limits and expenditure, but considerable benefit is hidden in their qualitative use.
- Qualitative considerations may suffice to approximate causal answers.

Causal questions drive most scientific reasoning. This should entail plenty of causal analyses, but clinical psychology often avoids causality because the established gold standard, a randomized controlled experiment or trial (RCT), is in many cases infeasible. Although we cannot or should not manipulate variables such as gender, traumatic events, personality traits and other constructs, their effects on clinical outcomes must be investigated to inform prevention, intervention, policies, theories and further research.

## The Specific Problem of Causality in Observational Studies

The methodological toolbox has been greatly expanded. It now offers approaches to causal answers in non-randomized studies (Greenland, 2017). These new tools mainly address the *specific* problem of causality: Without randomization, a binary factor **X** (group comparison, e.g., with and without a bipolar disorder diagnosis) and outcome **Y** (e.g., amount of substance use) often have *shared causes*, **Z** (e.g., parental mental health), that are out of experimental control and cause bias in an estimate of the average effect of **X** on **Y.** In linear models and for just a single **Z**, this bias is the product of the effect of **Z** on **X** and **Y**, meaning that it equals α_1 *_ α_2_, where α_1_ denotes the effect of **Z** on **X**, and α_2_ the effect of **Z** on **Y** (e.g., Gelman & Hill, 2007, Chapter 9). This simple formula implies that

- bias occurs only if α_1_ ≠ 0 *and* α_2_ ≠ 0
- the direction of bias just depends on the *signs* of α_1_ and α_2_. If they are equal, bias is upward, otherwise downward.
- bias is small if *either* is small

These properties generalize to non-linear relations and any distributions of **Y** and **Z** and to multiple **Z** that are independent or positively inter-related (Groenwold, Shofty, Miočević, van Smeden, & Klugkist, 2018; Pearl’s “adjustment formula” is the most general expression; Pearl, 2009). We refer to the above as the *basic confounding relation*.

Experimental control and randomization together disconnect all *confounders* **Z** from **X** and thus eliminate *confounding bias*. Otherwise, **X** is just *observed*, and in life-sciences like clinical psychology the number of natural causes of an **X** might be vast. The new methodical tools try to unravel the **X-Y** relation in an imaginary world in which **X** (or **Y**) was independent of **Z** and thus simulate what *changing* (rather than observing) **X** would do with **Y** (“do(**X**),” Pearl, 2009). The new methods mimic what might be observed if **X** were changed, but unlike real-world change experiments where **X** is isolated, their use requires an explicit understanding of the relationships between variables **Z** and **X**. Likewise, during their elaboration it has been stressed that one must consider *how* an **X** is to be changed because this may make a large difference (Greenland, 2005a). For example, just stopping drug use might even worsen an outcome if an intervention does not address factors like stress coping, a putative cause of drug use. In this sense, the new methods complement randomized experiments and RCTs through the more explicit need to go beyond a single **X**, thus to move from “causal description” to “causal explanation” (Johnson, Russo, & Schoonenboom, 2019). For other (non-specific) sources of bias like selection and measurement error that also effect the results of randomized studies, see the Supplementary Materials.

Instead of making use of the new methodological toolbox to approach causal answers in observational studies, clinical psychology was dominated by the “mantra” that “correlation is not causation” (Pearl & MacKenzie, 2018, back of the book). For a historical account on how this stance has emerged through the statistical pioneer Karl Pearson, who had considered causality to equal perfect (deterministic) correlation, see Pearl and MacKenzie (2018).

## Aim of This Paper

Some papers have already introduced tools from the new methodical box in (clinical) psychology and summarized the meanwhile vast literature on them (Dablander, 2020; Marinescu, Lawlor, & Kording, 2018). However, these have mainly focussed on quantitative approaches in a discipline where methodical causal thinking is new and, thus, requires qualitative guidance beforehand. One such instance is that psychology needs not only to overcome “retreating into the associational haven” (Hernán, 2005), but also immunization against overconfidence (Greenland, 2012) in novel methods. Overconfidence mainly concerns the quantitative and highly formal methods, because the mathematical sophistication in these easily obstructs the sight for hidden assumptions and over-simplification through translation into mathematics (Greenland, 2012, 2017; VanderWeele, 2016). Costs of using these methods also include learning and conducting them (which is error-prone) and the further degrees of freedom in analysis through their use which promotes p-hacking. We argue that qualitative approaches as exemplified in this article are easier to access and invite more debate and refinement on them and should at least inform the decision of using a particular quantitative method. We focus on a few causal conceptions that we believe are most illustrative for causal quests: the above basic confounding relation (1), counterfactuals (2), popular qualitative considerations (3) and directed acyclic graphs (DAGs) (4).

The following figure illustrates the scheme by which we describe how qualitative approaches may guide a causal quest.

**Figure 1:**
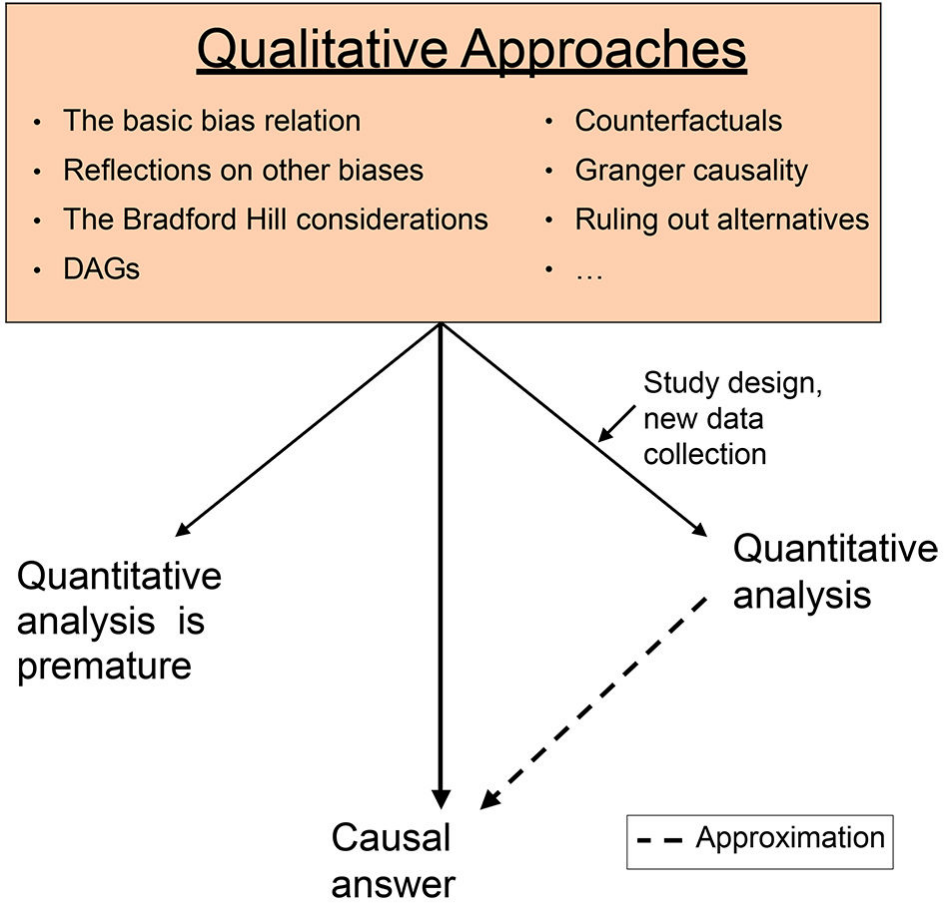
Scheme of Qualitative Approaches Guiding Causal Quests *Note.* These might be sufficient for overall causal answers, give rise to designing a new study and/or quantitative analysis, or suggest that such analysis is premature. The basic bias relation, counterfactuals and DAGs belong to the new toolbox of causal methods.

## Qualitative Approaches

### Gender Effects and the Basic Bias Relation

The effects of gender (biological sex) may play an important role for the development and maintenance of mental disorders. If they exist to considerable extent, they contribute to explaining the different aetiology of disorders that are more prevalent in females (e.g., internalizing disorders such as depression) and males (e.g., externalizing disorders such as substance use disorders). This is because gender may also affect many putative aetiological factors (e.g., response styles such as rumination; Johnson & Whisman, 2013; which, in turn, may influence the onset of disorders; Emsley & Dunn, 2012).

But is the causal wording “effect” warranted here? With the basic bias relation, we are equipped to ask: Are there shared causes of gender and a disorder **Y**? If it holds true that gender is largely random in the sense that it depends only on factors that do not also affect the disorder (Scarpa, 2016, and references therein), then no confounding bias is expected. If such factors exist (e.g., environmental pollution; Astolfi & Zonta, 1999) but affect **Y** only weakly, they may be neglected since the bias through them should be small. If bias from other sources is also negligible like selection and measurement, a causal conclusion seems informed.

### Upward Bias Through Confounders That Affect X and Y With the Same Sign

In the presence of reliable associational results, the basic bias relation can be applied well beyond gender effects. If there is at most a *weak* association between an **X** and a **Y**, and assuming that the common causes of **X** and **Y** affect both positively or both negatively (and are unrelated or positively inter-related), bias should be *upward*. Hence, the effect of **X** on **Y** should be smaller than the association and, thus, be absolutely small (and probably negligible). For example, the relatively weak and often inconsistently reported association between anxiety and alcohol use might be explained by genetic and personality factors increasing the risk for both (Schmidt, Buckner, & Keough, 2007). Such risk increasing may frequently apply: psychopathology in parents, genetic factors, stable personality traits, stressful life events and prior mental disorders are factors that might all affect disorders positively and be positively inter-related (Uher & Zwicker, 2017). However, with a larger number of shared factors, the probability rises that some have negative relations, but if these are few and unlikely to dominate bias (because their effects on **X** *and* **Y** are not very large as compared to those of the other factors), a researcher may still use the consideration.

### Counterfactuals and a Defendable Assumption on Them

The above gender example brings up an important limitation yet in the standard “counterfactual” definition of a causal effect. Biological sex cannot be entirely changed (beyond transsexual transformation) or imagined to be changed, but social aspects of gender can (Glymour & Glymour, 2014).

Imagining a person under an alternative **X** condition is called *counterfactual* and defines an effect as the amount of change in **Y** if **X** is changed from one value to another (if this equals zero, there is no effect). Consider the putative effect of childhood trauma (CT) on depression (DE). Yet the idea of counterfactuals points out that “the effect” is imprecise since there are actually two counterfactuals and associated effects: a) trauma *experience* in individuals who actually do *not* experience trauma and b) trauma *recovery* in those who actually had experienced a trauma (but do not recover). Just referring to “the effect” denotes the *total* effect, which means that we imagine *both* changes at once (Pearl, 2009). Such a summary appears pointless in clinical psychology, at least if one aims to keep aetiology and persistence/maintenance apart which seems important since in many cases, different factors seem to be involved in the onset versus the persistence of mental disorders (McLaughlin et al., 2011).

The effect of *experiencing* a CT is, in principle, subject to a prevention RCT, but such studies would be highly ineffective. This is because CT prevention will never succeed among all individuals and is unethical if the control group is deliberately exposed to CT although exposure (and associated harm) could have been prevented. The effect of *recovery* from a trauma on the other hand; i.e., of successful intervention, can in principle be investigated in an RCT, but only with regard to specific *consequences* of CT. This not only heavily depends on what is meant with “consequences” (e.g., distress, symptom onset, incidence of a diagnosis) and the mode of intervention, it is confounded with the aim of investigating the recovery effect (Greenland, 2005a).

At least for onset, “target trials” (here prevention trials) may be an effective further tool to clarify what a counterfactual specifically means (VanderWeele, 2016). A *target trial* is an ideal trial (or experiment) the data of which would provide the desired causal answer. It clarifies qualitatively what we *would* require, what we cannot do, but what we can anyway *imagine* (Lewis, 1973; Pearl, 2013), including the target population to infer on.

For a conclusion on the *existence* of either effect, crude estimates of counterfactual depression rates (generally mean outcomes) among those with and without CT, respectively, are necessary. If we know empirically that, say, 5% of those without CT develop depression later in life, and we assume that the experience of CT in all the observed individuals would have increased this rate (i.e., the counterfactual rate is >5%; probably few clinical psychologists would doubt this), the conclusion that *CT experience increases the risk for depression* is valid. Likewise if, say, 10% of those with CT have depression later on, we may conclude that an intervention *decreases* the rate provided that we are willing to assume that the intervention would achieve a rate below 10%.

This line of qualitative argument determines the “target quantity” (Petersen & Van der Laan, 2014) one wishes to estimate. It may also trigger other considerations like *substituting* unknown counterfactual depression rates from other, “analogous” (Hill, 1965) studies. For trauma experience, a sample of children traumatized by war may be used and for recovery, a sample of traumatized, untreated but resilient children.

### Granger Causality

Imagining counterfactual states of brains in Neuroscience and Neuroimaging research seems meaningful, but in associated longitudinal studies there is a shortcut to the specific causal problem of common causes hidden in the term “Granger causality” (Friston, Moran, & Seth, 2013). Originally, the term states that, given “all the information in the universe up to time t” (Eichler & Didelez, 2010), and provided that the prediction of **Y** at time t + 1 is worse if an **X** at any time up to t is disregarded, then this prior **X** is a cause of **Y** (Granger, 1969). Although equivalent with the counterfactual definition, Granger causality has been frequently mistaken as only referring to *observed* **X** variables (Eichler, 2012; Eichler & Didelez, 2010) or even just a time-series of a single **X** (Marinescu et al., 2018). This downgrades the conception into a heuristic for practical use with the easily wrong qualitative suggestion that adjustment for common causes has been sufficient. Researchers who use it must be aware of the basic bias relation indicating that they play into their own hands if they ignore unobserved common causes that effect **X** and **Y** with the same sign. These may include variables that have occurred before study onset. Generally, collecting *big data* like thousands of voxels in a brain scan is no substitute for thoughtful reflections on the processes beyond the data that any defendable causal analysis relies on (Pearl & MacKenzie, 2018).

In the Supplementary Materials we briefly discuss other popular and, mostly long-used approaches: multimethod evidence, mixed methods research and ruling out alternatives.

### Directed Acyclic Graphs

So far, we have only addressed direction of bias but not when and how bias can be removed. In the Supplementary Materials, we revisit the example of the effect of CT on DE to outline the qualitative answers that the qualitative method of DAGs provides, including the subsequent study design and analysis that a particular DAG model may give rise to. The example uses a model with four common causes and causal relations among them. It reveals that adjustment for them is possible in subsequent quantitative analysis (whereby one shared cause does not require adjustment).

Importantly, DAGs may include effects of unchangeable factors like “socio-economical family status” in the example where the counterfactual conception of an effect does not apply. The conception, however, may be extended to include other actors than humans who could change an **X** (Bollen & Pearl, 2013). Sometimes such an actor is difficult to name let alone to translate into a mathematical model, wherefore instances like “socio-economical family status” are more suited “to describe something as a cause” than to “reasonably define a quantitative causal effect estimand” (VanderWeele, 2016).

### Qualitative Assumptions May Make Quantitative Approaches Seem Premature

In contrary to the above instance, a DAG might reveal that bias can *not* be fully eliminated, or leave open whether an adjustment decreases or increases bias (Morgan & Winship, 2014, Chapter 3). The practical utility of DAGs for quantitative analysis rises with fewer variables in them and the number of causal relations that can be assumed not to exist (Greenland, 2017). However, setting up a DAG model should reveal this. Per se, a DAG renders all associated assumptions transparent and invites for debate and refinement on them (the reader might ask herself if this happens with the figure in the Supplementary Materials).

Anyway, controversy on a model might be so large that grounding a study and quantitative analysis on it appears unwarranted (Petersen & Van der Laan, 2014). Also, if the number of potential common causes is large and there is no way to prioritize them for reducing bias, quantitative analysis seems premature. Instead, more research is required beforehand to set up a defendable DAG. An example is the effect of internalizing symptoms on substance use where common causes may include a variety of genetic, parental, childhood, personality and environmental factors, as well as all sorts of individual variables related to neurobiological, cognitive and emotional processes (Pasche, 2012).

## Conclusions

No method can fully cover all aspects of causality across research fields and specific applications, especially in a life science as complex as clinical psychology (Greenland, 2017), and “there is no universal method of scientific inference” (Gigerenzer & Marewski, 2014). Likewise, a causal query can never be fully objective, because it always involves assumptions beyond the data (Greenland, 2005b). In sharp contrast, researchers tend to “mechanizing scientists’ inferences” (Gigerenzer & Marewski, 2014) and downgrade methods from tools for thoughtful cooperation between methodologists and substantive experts (Höfler, Venz, Trautmann, & Miller, 2018) into empty rituals (Gigerenzer, 2018).

In this article, we have outlined some qualitative approaches through which one may approach a crude causal answer on an average effect, plan a quantitative analysis or unravel that any analysis is currently infeasible. In fact, any causal quest must start with qualification because otherwise it would be just a mechanical exercise. The qualitative conceptions outlined here are meant as provisory heuristics that must not be ritualized but should be taken as invitations for refinement and adjustment to any particular application.

Above all, the two possible errors in causal conclusions should guide causal quests and the decision on whether the use of a highly formal method pays off (Greenland, 2012): false positive and false negative. Statistical decision theory provides the framework to formalize the balance between false positive and false negative causal conclusions. It states that the better decision is the one with the lower expected costs (Dawid, 2012).

Thoughtful causal quests are essential for explaining why phenomena occur the way they do and in providing levers through which things could be changed, for instance, in preventing disorders and improving life. Assessing causality is complex, demanding and ambivalent, but so is science. However, it makes use of the natural capacity of causal modelling which is deeply grounded in us human beings and structures how we view the world (Pearl & MacKenzie, 2018).

## Supplementary Materials

The supplement provides additions to the paper, namely other sources of bias than confounding, and futher popular approaches to causality besides those from the new toolbox and Granger causality. Besides, it addresses the example of the effect of childhood trauma (factor X = CT) on depression (outcome Y = DE) using a DAG (directed acyclic graph) model on common causes and subsequent study design and data analysis the model gives rise to (for access see Index of Supplementary Materials below).

10.23668/psycharchives.4838 Supplement 1 Supplementary materials to "Qualitative approximations to causality: Non-randomizable factors in clinical psychology" [Additional information]

HöflerM.
TrautmannS.
KanskeP.
 (2021). Supplementary materials to "Qualitative approximations to causality: Non-randomizable factors in clinical psychology"
[Additional information]. PsychOpen. 10.23668/psycharchives.4838
PMC966712736397960
