# Supplementary material for: Qualitative Approximations to Causality: Non-Randomizable Factors in Clinical Psychology
Source: Clin Psychol Eur. 2021 Jun 18;3(2):e3873. doi: 10.32872/cpe.3873 (PMC9667127; doi:10.32872/cpe.3873)
Supplement: Supplement 1 [file cpe-03-3873-s01.pdf]

Supplementary material to the article "Qualitative approximations to causality: non-randomizable factors in clinical psychology"

Michael Höfler, Sebastian Trautmann, Philipp Kanske

Appears in *Clinical Psychology in Europe*, 2021, Vol. 3(2), Article e3873,

https://doi.org/10.32872/cpe.3873

This online supplement provides some additions to the paper, namely other sources of bias than confounding, and other popular approaches to causality besides those from the new toolbox and Granger causality. It also elaborates on the example of the effect of childhood trauma (factor **X** = CT) on depression (outcome **Y** = DE) using a DAG (directed acyclic graph) model on common causes and subsequent study design and data analysis the model gives rise to.

1. Other sources of bias that also affect randomized studies

Randomized experiments and RCTs are also prone to other data-generating mechanisms that might distort causal analysis (Hernán et al., 2008). Such mechanisms, however, are *not specific* for causal quests but also bias estimates of associations. They include selection, measurement error in **Y** and non-compliance with the compared **X** conditions. Observational studies may yield similar results especially if assignment to **X** is largely independent of **Y** (Rosenbaum, 2010; Shadish et al., 2002), or if common causes are adjusted for (Anglemyer et al., 2014). If this is not the case, total bias might be even larger in randomized studies; for instance, if selection bias (in randomized clinical studies) dominates confounding bias (in

non-randomized general population studies, Greenland, 2005, 2012; Lash et al., 2009; Mansournia et al., 2017).

Consider *selection bias* in clinical studies. This occurs, for instance, if treatment seeking is related with the treatment effect of interest. It generally happens if whatever **X** and **Y** are both related with being selected into a study (through helpseeking). This mechanism became famous as "Berkson's bias" for the potential to create fully spurious comorbidity and is nowadays (within the framework of the below described DAGs) referred to as "conditioning on a collider" (Elwert & Winship, 2014; Höfler & Trautmann, 2019). For example, an association between years of education and severity of depression is expected in a clinical sample just because both higher education and disorder severity are associated with seeking treatment (Magaard et al., 2017).

2. Other qualitative methods

Multi-method evidence

A good advice is not to draw causal conclusions from just a single study, especially if studies with different methods (e.g. cell and animal studies, neuroimaging, self-reports) are available (Greenland, 2017) with *differently* biased estimates, because the study designs open the door for sources of bias to stream in differently (confounding, selection, measurement, non-compliance, etc.; Greenland, 2012). Then *convergent* evidence for an effect can hardly be explained by *common* bias (bias that equally occurs in all kind of studies; Campbell & Fiske, 1959). However, different biases could distort the estimates in the same direction. For instance, placebo treatment effects have been argued to be upward biased both due to selection into a clinical sample and reporting **Y** (Hróbjartsson et al., 2011). Thus, studies in

both clinical (biased through both) and general populations (biased only through reporting) might be inflated, in which case the common bias argument fails.

On the other hand, the argument can be strengthened through adding some of the nine Bradford Hill considerations that Hill had once formulated inspired by the historical controversy on the effect of smoking on lung cancer (Hill, 1965). Such are: “strength of association” (as above) and “plausibility”: there is a (biological) model that explains an effect. The latter evaluates a (maybe quantitative) finding or putative conclusion through the ability to link it to substantive knowledge. However, which finding and which knowledge weighs how much is strongly context-dependent (Höfler, 2005), and different integrations of “ragged evidence” might appear plausible but yield different conclusions (Greenland, 2012).

Mixed methods research

Recently, similar proposals have been made under the term of “mixed methods research”. “Mixed methods“ call for study type “pluralism“ and urges researchers to reflect on different levels (persons, populations, time, situations, factors and outcomes) where causal effects may occur equally or differently (Johnson et al., 2019, and references therein).

Ruling out alternatives

Another popular instance of considering more background knowledge than addressed in an analysis is ruling out alternatives to **X** causing **Y** (Greenhouse, 2009). Statistically, an **X-Y** association may be explained, among others, by shared causes, shared measurement error, selection bias, and inverse causation (**Y** causing **X**) (Maclure & Schneeweiss, 2001; Pearl, 2009).

3. DAGs and the effect of childhood trauma (CD) on depression (DE)

A directed acyclic graph (also “causal diagram”) displays arrows that encode whether a variable is assumed to affect another (arrow) or not (no arrow) (Pearl, 1995, 2009). An entire graph expresses *qualitative* assumptions on common causes and causal dependencies among them. After setting up a DAG, it is evaluated with regard to study design and quantitative analysis it may suggest (see below). A DAG is *non-parametric*; that is, all mathematical theorems apply independent of how the variables are scaled and distributed and according to what mathematical function they affect one another. A diagram must be *complete* with regard to the shared causes of **X** and **Y**. The arrows in the graph code assumptions on *direct effects*; for instance, we suppose that “socio-economical family status” affects depression directly (and indirectly via “risk environment”).

Different and much recommended introductory accounts for the field are provided by Dablander (2020) and Rohrer (2018). Here, we illustrate how a DAG accounts for *bias due to confounding*. Note that also the the bias sources of measurement (Hernán & Cole, 2009), non-compliance (Morgan & Winship, 2014; ch. 9), selection (Bareinboim & Pearl, 2016; Elwert & Winship, 2014) and missing data (Thoemmes & Mohan, 2015) can be addressed with DAGs, while revealing whether and how an effect can be estimated without bias.

For the effect of CT on DE we suppose just *four common causes* to demonstrate the use of the method rather than to provide an exhaustive account of all variables that are theoretically plausible and in line with the evidence: parental psychopathology (PP; Hankin, 2015; Lizardi & Klein, 2000), socio-economical family status (SFS; Freeman et al., 2016; Freisthler et al., 2006), risk environment (RE, e.g. living in a dangerous neighbourhood; Coulton et al., 2007)

and genetic factors (GF; Dunn et al, 2015; Peyrot et al., 2014). Figure 1 shows the model including the supposed effects of these factors on one another:

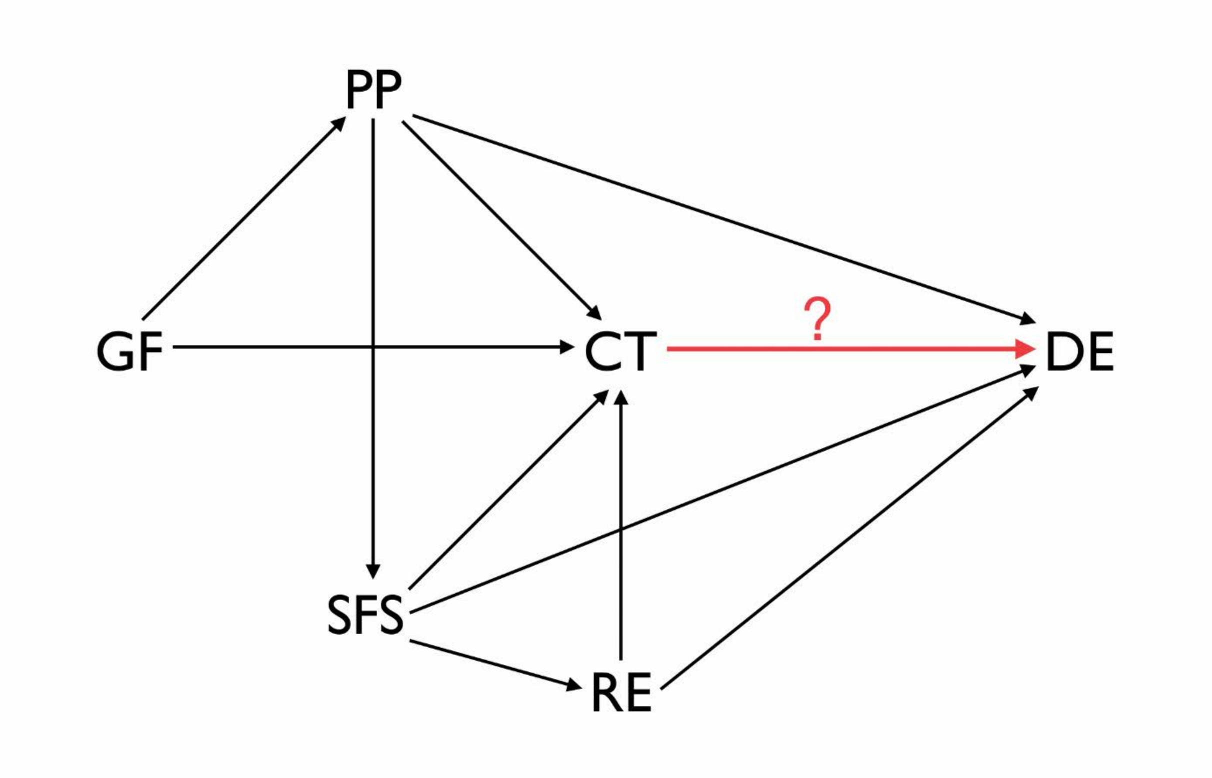

Legend to Figure 1:

DAG model of common causes of childhood trauma (CT) and depression (DE) and the causal relations between them. Assumed common causes are parental psychopathology (PP), socio-economical family status (SFS), risk environment (RE) and genetic factors (GF)

The factor of interest, CT is supposed to be influenced by parental psychopathology (PP), socio-economical family status (SFS) and risk environment (RE). CT may be affected by these variables through whatever function, f_{CT}, CT = f_{CT}(PP, SFS, RE, ϵ_{CT}). ϵ_{CT} is a summary of all other variables that influence CT. These are not visualized in the graph for parsimony since they have no effect on DE and, thus, on how adjustment should be done. Note that genetic factors (GF) also influence CT, but only indirectly through the path GF → PP → CT.

DAGs encode *direct* effects in terms of the other variables that the graph contains. PP, for example, may actually affect CT through other variables, but if these do not have causal connections with DE, these can be omitted as well.

Now, the “backdoor criterion” (Pearl, 1995) provides an algorithm to identify paths in the model that bring about non-causal associations between CT and DE and are to be eliminated. These are called “backdoor paths“, a backward path must contain an arrow into CT and a backward connection into DE.

In the given example, there are seven backdoor paths:

(1) CT ← PP → DE

(2) CT ← PP ← GF → DE

(3) CT ← SFS → DE

(4) CT ← RE → DE

(5) CT ← SFS → RE → DE

(6) CT ← PP → SFS → DE

(7) CT ← PP → SFS → RE → DE

Furthermore, a backdoor path must also be "collider-free" to bring about non-causal association. This is because a common consequence (“collider”) of two factors does not cause an association between these factors — unless the common consequence is wrongly adjusted for (Elwert & Winship, 2014). In the example, a variable outside the model, school grades (SG), could be a common consequence of CT and SFS: CT → SG ← DE. Adjusting for SG would yield an otherwise not present CT-SFS association and, in turn, new backdoor paths and additional bias.

Now, the backdoor-criterion states that we have to identify a subset of confounders, that, if adjusted for, “blocks“ paths 1-7 such that, given the subset, the association between CT and

DE is independent of the omitted confounders. This means that each backdoor path must lead through at least one variable of the subset. Here, only the sets (PP, SFS, RE) and (PP, SFS, RE, GF) fulfil this criterion. Thus, we don't have to consider GF if we take PP, SFS and RE into account.

Importantly, the results of effect quantification may yet vary strongly across different specified DAGs. Different DAGs may appear similarly plausible, in which case researchers are well advised to collect *all variables* that are necessary for the different adjustments that the different DAGs call for. If one is lucky, however, sensitivity on this level is small because the different DAGs address the same key features of bias, although each model may just be a crude map of reality (VanderWeele, 2016).

4. Study design

The model tells us that we have to design a study that collects data not only on CT and DE (while establishing temporal sequence, the prerequisite for causation, ideally in a prospective study), but also on PP, SFS and RE. This is the essence of designing an observational study for causal inference (Rosenbaum, 2010; Shadish et al., 2002). In the example, PP, SFS and RE are summary variables. For adjustment to be sufficient, these constructs should be completely covered through a collection of variables that addresses all of a construct's aspects, and these must share the same causal relations (Ramsahai, 2012). For example, RE should include information on local crime rates, regional conflicts, air pollution and lack of infrastructure. To address PP sufficiently, parents should be comprehensively diagnosed. Moreover, to succeed completely in adjustment, the confounders have to be measured without error. Otherwise "residual bias" is expected (Morgan & Winship, 2018). For example, PPP

assessment should use the best available instrument. Lastly, to keep bias due to selection small, a sample should be drawn from a source population that resembles the target population in the parameter of interest, here the magnitude of the effect of CT on DE (Elwert & Winship, 2014). Again, this requires assumptions beyond the data, and these can be expressed with a DAG (Bareinboim & Pearl, 2016).

5. Adjustment after data have been collected

For the present purpose, we provide a brief summary with principal guidance on the adjustment methods because their details are vast and better explained with data. A comprehensive account with rich citations of original work is provided by Morgan and Winship (2014, chapters 5-7).

The simplest adjustment method to estimate the average treatment effect, **ATE**, is jointly regressing **Y** on **X** and the chosen **Z** variables in order to remove the **Z-Y** relations when comparing the **X**-groups. The method is very crude and may be pretty ineffective in balancing, in the example, individuals with and without CT with regard to the distributions of PP, SFS and RE. It performs increasingly poorly the more the effect of CT on depression varies across individuals. Heterogeneity in effects appears at least plausible for many if not most mental disorders and their aetiological factors. Regression may also work not well if the model does not fit the data or makes wrong assumptions on the distribution of errors. However, better fitting models (like generalized linear models) can be used. Besides, adding interactions between CT and effect-modifyers (which may differ from PP, SFS and RE) could capture at least some important features of effect-heterogeneity. Such a model also serves estimating potential outcomes: Given whatever value of whatever **Z**, the regression equation predicts the average outcome **Y** under **X** = 1 and **X** = 0 and, thus, the group difference in **Y** given **Z** (i.e., in individuals with such a **Z** value). This works for every combination of **Z**

values, and averaging over these yields an estimate of **ATE** like the average effect of CT on depression. Alternatively, the individuals may be differently weighted (using the propensity score, see below), such that **ATT** or **ATC**, respectively, are estimated: the effect of CT removal or CT experience, respectively.

Propensity-score methods instead focus on removing the **X-Z** relations. They firstly require a model on group assignment; that is, on the probability that **X** equals 1 given **Z**. This probability is called propensity score, ps. If ps is known and adequately adjusted for, ps is sufficient for unbiased effect estimates, the distinct **Z** variables do not need to be taken into account anymore (Rosenbaum & Rubin, 1983).

In the example, ps is the probability that an individual is traumatized in childhood, given her values in PP, SFS and RE (irrespective of whether an individual has truly experienced CT or not). ps is estimated for each individual with a model, often through logistic regression of P(**X** = 1) on **Z** yielding model-predicted probabilities of **X** = 1 given **Z**. Importantly, the model must be true in describing the actual assignment process. For instance, if PP and SFS interact in affecting the CT risk on the logistic scale, this interaction must be included in the regression equation. Otherwise **X** is not fully disconnected from **Z** and, again, residual confounding occurs. Uncertainty in model selection can be addressed by averaging an individual's ps across models that similarly fit the data. This is prevalent if the number of **Z** variables is large and in small samples and can be handled with "random forests" if the sample proportion of X = 1 ranges, say, between 30 and 70 percent. (A random forest is a collection of possible models, each of having the shape of a "tree" that predicts the average **Y** in each "terminal node" of the tree. Each such node results from a series of binary splits according to values of **Z** variables that bring about the largest difference in **Y** (Strobl et al., 2009)).

After a model has been fitted, the range and distribution of ps values in both **X** = 0 and **X** = 1 must be inspected. It may turn out that the groups have different ps ranges, which may indicate that, for example, some **X** = 1 individuals have no "twin" in **X** = 0 with regard to ps (and, thus, one or more **Z** variables). Such a finding may suggest that, for these individuals, **X** = 0 is not a meaningful counterfactual. This should, however, rarely happen if the model in the underlying DAG is sound. Otherwise, individuals that lack twins in the other group should be omitted from analysis and the analysis be restricted to the "region of common support". In consequence, the inferential population is limited to individuals with ps in this region. In the example, the data might contain individuals with low RE, low PP and high SFS only in the non-CT group. Omitting these would not allow including them in a causal conclusion.
Another practical issue is ensuring whether ps sufficiently summarizes the **Z** variables; that is, whether **X** and each **Z** are independent given **Z**.

Once ps is computed and the points discussed above are addressed, there are several approaches that use ps to balance **X** = 0 and **X** = 1. Unlike parametric regression methods statistical matching methods draw each individual from one group with its observed **Y** value, calculate the difference with each observation in the other group that is similar in ps, then average across these "twins" and finally average across individuals. These procedures are non-parametric because they do not rely on assumptions on the distributions of **Y** in **X** = 0 and **X** = 1.
Matching can be done in individuals with **X** = 0, e.g. adjusting individuals with CT to those without CT (**ATC** estimation), or within **X** = 1 individuals, adjusting individuals with CT to those without CT (**ATT** estimation). Averaging **ATC** and **ATT** (weighted by the number of individuals with CT and without CT, resp.) yields **ATE**.

Specific matching algorithms differ in how they operationalize “similar” and weight the observations according to the magnitude of similarity (e.g. “kernel matching”, “genetic matching” and “optimal matching”). Unfortunately, the literature is inconsistent in what method works best. Therefore, the recommendation is using a range of methods to ensure that a particular conclusion is not due to the specific method used. Of course, all results then have to be reported and the exact analytic plan should be registered beforehand to prevent p-hacking.

Finally, *doubly robust estimation* methods (“inverse-probability-weighted regression adjustment” and “augmented inverse-probability”) combine the approaches of eliminating the **Y-Z** and **X-Z** relations. They do this by jointly a) regressing **Y** on **X*Z** (and the main effects of **X** and **Z**) *and* b) weighting the individuals with functions of the propensity score. They are called doubly-robust because their bias in estimating the **X-Y** effect is the product of the biases in a) and b): If one bias is zero (small) the total bias is zero (small). Thus, one has two attempts to succeed (and each attempt is robust against wrong assumptions in the other). Today, various software packages including R, Python, Stata and SPSS include a range of these methods. However, care in their use must be taken since their implementation might differ what may cause unwarranted variation in results.

Finally note that DAGs give rise to two *other quantitative methods* to address bias due to confounders: a) “Mechanism-based” unravels an **X-Y** effect into direct and indirect effects that may be less confounded than the total effect (Morgan & Winship, 2014, chapter 10).
b) The “instrumental variables” approach is often used to model non-compliance in **X** in a treatment or experimental study. An *intended* randomized treatment may serve as an “instrument”, **I**, for estimating the effect of treatment according to the protocol (= **X**, what is confounded) as if there was perfect compliance. Under certain assumptions, the **X-Y** effect

can be calculated from the **I-X** and **I-Y** associations (which may be less confounded), but possibly only in a restricted population (Morgan & Winship, 2014, chapters 10 and 9., resp.). Also note how a DAG relates to observation and thus be empirically tested: It predicts a set of associations through pairs of variables that are causally or non-causally linked (e.g. due to common causes). Each of these predicted associations might be found in the subsequent study or not and, thus, invite model modification.

References

Anglemyer, A., Horvath, H. T., & Bero, L. (2014). *Healthcare outcomes assessed with observational study designs compared with those assessed in randomized trials*. Cochrane Database Syst Rev *4*, p. Mr000034 https://doi:10.1002/14651858.MR000034.pub2

Bareinboim, E., & Pearl, J. (2016). Causal inference and the data-fusion problem. *Proceedings of the National Academy of Sciences*, *113*(27), 7345-7352. https://doi.org/10.1073/pnas.1510507113

Campbell, D. T., & Fiske, D. W. (1959). Convergent and discriminant validation by the multitrait-multimethod matrix. *Psychological Bulletin*, *56*(2), 81–105. https://doi.org/10.1037/h0046016

Coulton, C.J., Crampton, D.S., Irwin, M., et al (2007). How Neighborhoods Influence Child Maltreatment: A Review of the Literature and Alternative Pathways. *Child Abuse & Neglect*, *31*(11–12), 1117–42. https://doi:10.1016/j.chiabu.2007.03.023

Dablander, F. (2020). An Introduction to Causal Inference. *PsyArXiv*. February 13. d https://doi:10.31234/osf.io/b3fkw

Dunn, E. C., Brown, R. C., Dai, Y., Rosand, J. et al. (2015). Genetic Determinants of Depression: Recent Findings and Future Directions. *Harvard Review of Psychiatry, 23*(1), 1–18. https://doi:10.1097/HRP.0000000000000054

Elwert, F., & Winship, C. (2014). Endogenous Selection Bias: The Problem of Conditioning on a Collider Variable. *Annual Review of Sociology, 40*, 31–53. https://doi: 10.1146/annurev-soc-071913-043455

Freeman, A., Tyrovolas, S., Koyanagi, A., et al. (2016). The Role of Socio-Economic Status in Depression: Results from the COURAGE (Aging Survey in Europe). *BMC Public Health 16*, 1098. https://doi:10.1186/s12889-016-3638-0

Freisthler, B., Merritt, D. H., & LaScala, E. A. (2006). Understanding the Ecology of Child Maltreatment: A Review of the Literature and Directions for Future Research. *Child Maltreatment, 11*(3), 263–80. https://doi.org/10.1177/1077559506289524

Greenhouse, J. B. (2009). Commentary: Cornfield, Epidemiology and Causality. *International Journal of Epidemiology 38*, 1199–1201. http://doi:10.1093/ije/dyp299

Greenland, S. (2005). Multiple-bias modelling for analysis of observational data. *Journal of the Royal Statistical Society A, 168*(2), 267–291. https://doi.org/10.1111/j.1467-985X.2004.00349.x

Greenland S. (2012). Causal inference as a prediction problem: Assumptions, identification and evidence synthesis. In: Berzuini C, Dawid P, Bernardinelli L (eds). *Causality: Statistical Perspectives and Applications*. Hoboken, NJ: Wiley:43–58.

Greenland, S. For and Against Methodologies: Some Perspectives on Recent Causal and Statistical Inference Debates. (2017). *European Journal of Epidemiology, 32*(1), 3-20.

Hankin, B. L. (2015). Depression from Childhood through Adolescence: Risk Mechanisms across Multiple Systems and Levels of Analysis. *Current Opinion in Psychology, 4,*13–20. https://doi:10.1016/j.copsyc.2015.01.003

Hernán M. A., Alonso, A., Logan, R. et al. (2008). Observational studies analyzed like randomized experiments: an application to postmenopausal hormone therapy and coronary heart disease. *Epidemiology, 19*(6), 766–79. https://doi:10.1097/EDE.0b013e3181875e61

Hernán, M., A., & Cole, S.R. (2009). Invited Commentary: Causal Diagrams and Measurement Bias, *American Journal of Epidemiology*, *170*(8), 959–962, https://doi.org/10.1093/aje/kwp293

Hill, A. B. (1965). The Environment and Disease: Association or Causation? *Proceedings of the Royal Society of Medicine, 58*(5), 295–300. https://doi.org/10.1177/0141076814562718

Höfler, M. (2005). The Bradford Hill considerations on causality: A counterfactual perspective. *Emerging Themes in Epidemiology, 2*, 11. https://doi:10.1186/1742-7622-2-11

Höfler, M., & Trautmann, S. (2019). Letter to the editor: When does selection generate bias in clinical samples? *Journal of Psychiatric Research, 116,* 189-190. https://doi:10.1016/j.jpsychires.2019.02.010

Hróbjartsson, A., Kaptchuk, T. J., & Miller, F. G. (2011). Placebo effect studies are susceptible to response bias and to other types of biases. *Journal of clinical epidemiology*, *64*(11), 1223–1229. https://doi.org/10.1016/j.jclinepi.2011.01.008

Johnson, R.B., Russo, F., Schoonenboom, J. Causation in Mixed Methods Research: The Meeting of Philosophy, Science, and Practice. *Journal of Mixed Methods Research*. 2019;13(2):143-162.

Lash, T.L., Fox, M.P., Fink, A. (2009). *Applying Quantitative Bias Analysis to Epidemiologic Data.* New York, NY: Springer.

Lizardi, H., & Klein D.N. (2000). Parental Psychopathology and Reports of the Childhood Home Environment in Adults with Early-Onset Dysthymic Disorder. *The Journal of Nervous and Mental Disease*, *188*(2), 63–70. https://doi.org/10.1097/00005053-200002000-00

Maclure, M., & Schneeweiss, S. (2001) Causation of bias: the episcope. *Epidemiology*, *12*(1), 114–122.

Magaard, J. L., Seeralan, T., Schulz, H., & Brütt, A. L. (2017). Factors associated with help-seeking behaviour among individuals with major depression: a systematic review. *PLoS One.* 12(5):e0176730 https://doi:10.1371/journal.pone.0176730

Mansournia, M. A., Higgins, J. P. T., Sterne, J. A. C., & Hernán, M. A. (2017). Biases in randomized trials: a conversation between trialists and epidemiologists. *Epidemiology* *28*(1): 54–59. https://doi:10.1097/EDE.0000000000000564

Morgan, S. L., & Winship, C. H. (2014). *Counterfactuals and Causal Inference. Methods and Principles for Social Research.* 2nd edition. Cambridge, UK: Cambridge University Press.

Pearl, J. (1995). *Causal diagrams* for empirical research. *Biometrika*, *82*(4), 669-688. https://doi.org/10.1093/biomet/82.4.669

Pearl, J. (2009). *Causality, models, reasoning and infere*nce. 2nd edition. Cambridge, UK: Cambridge University Press.

Peyrot, W. J., Milaneschi, Y., Abdellaoui, A. et al. (2014). Effect of Polygenic Risk Scores on Depression in Childhood Trauma. *The British Journal of Psychiatry: The Journal of Mental Science, 205*(2), 113–19. https://doi:10.1192/bjp.bp.113.143081

Ramsahai, R.R. (2012). Supplementary variables for causal estimation In: C. Berzuini, P. Dawid, L. Bernardinelli (eds). *Causality: Statistical Perspectives and Applications* (pp. 218-233). New York, NY, USA: Hoboken: Wiley.

Rohrer, J. (2018). Thinking Clearly About Correlations and Causation: Graphical Causal Models for Observational Data. *Advances in Methods and Practices in Psychological Science 2018, 1*(1), 27 –42. https://doi.org/10.1177/2515245917745629

Rosenbaum, P.R. (2010). *Design of Observational Studies*. New York, NY, USA: Springer.

Rosenbaum, P.R.; & Rubin D. B. (1983). The Central Role of the Propensity Score in Observational Studies for Causal Effects. *Biometrika*, 70(1), 41-55. https://doi.org/10.1093/biomet/70.1.41

Shadish, W.R., Cook, T.D., & Campbell, D.T. (2002). *Experimental and Quasi-Experimental Designs for Generalized Causal Inference.* Boston, MA, USA: Houghton Mifflin Company.

Strobl, C., Malley, J., & Tutz, G. (2009). An introduction to recursive partitioning: Rationale, application and characteristics of classification and regression trees, bagging and random forests. *Psychological Methods, 14*(4), 323-348. https://doi.org/10.1037/a0016973

Thoemmes, F., & Mohan, K. (2015). Graphical Representation of Missing Data Problems *Structural Equation Modeling: A Multidisciplinary Journal 22*(4), 631–642. https://dx.doi.org/10.1007%2Fs10654-018-0447-z

VanderWeele, T. J. (2016). Commentary: On Causes, Causal Inference, and Potential Outcomes. *International Journal of Epidemiology*, *45*(6), 1809-1816. https://doi:org/10.1093/ije/dyw230
